# The Risk of Depression Among Burn Injury Survivors; A Systematic Review and Meta-analysis

**DOI:** 10.1007/s44197-025-00479-7

**Published:** 2025-11-21

**Authors:** Ali Mohamed Elameen, Asmaa Ali Dahy, Amany Attalah Gad

**Affiliations:** 1 Department of Plastic and Reconstructive Surgery, El-Sahel Teaching Hospital, Cairo, Egypt; 2 Department of Plastic and Reconstructive Surgery, Faculty of Medicine For Girls, Al-Azhar University, Gameat Al Azhar, Nasr City, Cairo, Egypt

**Keywords:** Burn, Depression, Depressive, Survivors

## Abstract

**Background:**

Depression is a prevalent psychological complication among burn injury survivors. Affected patients face increased risks of prolonged hospitalization, additional surgical and reconstructive interventions, higher healthcare utilization, and suicidal ideation. This systematic review and meta-analysis quantified depression prevalence by severity and examined factors associated with post-burn depression.

**Methods:**

We searched twelve databases from inception to 14 June 2023. We included clinical studies comparing the demographic, burn-related, or management-related factors between patients with depression and patients without depression among burn injury survivors.

**Results:**

Of the pooled 2,957 patients from ten articles, 36.8% experienced depression. Among those with depression, 33.1% had mild, 34.0% had moderate, and 10.8% had severe depression. Meta-analysis indicated an overall depression prevalence of 60.7% (95% confidence interval [CI]: 44.5–74.8%). Patients with total body surface area > 30% had 2.48 times higher risk of depression. Age, gender, marital status, burn type, burn degree, and burn site did not significantly influence risk of depression among burn injury survivors.

**Conclusion:**

Depression affects approximately two-fifths of burn survivors, with one-quarter experiencing moderate depression and one-tenth severe depression, especially in major burns. Systematic psychological assessment and standardized mental health interventions are warranted to optimize recovery and quality of life.

**Supplementary Information:**

The online version contains supplementary material available at 10.1007/s44197-025-00479-7.

## Introduction

 Burn is a worldwide health issue, significantly burdening healthcare facilities. Of note, approximately 11 million individuals suffer from burn injuries yearly, leading to over seven million living with disabilities and more than 180,000 deaths annually [1, 2]. Burn injuries are associated with debilitating sequelae on the physical health, psychological well-being, and quality of life of burned patients [3, 4]. Severe burns incur lifetime costs roughly five times higher than stroke, exceeding $211 million annually for US patients. The associated costs-disability care, caregiver burden, psychological trauma, and lost productivity impose significant economic and social strain on patients, families, and healthcare systems [5–7].

Burn injuries represent traumatic experiences that entail substantial psychological burdens, including impaired physical function, post-traumatic stress disorder (PTSD), altered body image, sleep disturbances, and depression [8]. The skin renders burn-related disfigurement particularly impactful, often leading to dysmorphic behavior and reduced self-esteem [9]. The psychological burden arises from both injury and resultant physical disabilities, including persistent scarring and contractures. These impairments limit functional capacity and social engagement, frequently resulting in social exclusion [4, 10]. This poses a more significant burden for burn care survivors and their families to cope with the challenging new life. This highlighted the urgent need to evaluate the psychological and mental status of burn injury survivors promptly to mitigate the negative consequences of burn [9, 11].

Depression is a common psychological complication among burn survivors, with an estimated incidence of 10–23% within the first-year post-injury, approximately three- to fourfold higher than in the general population. This risk may increase to 42% after two years, highlighting the necessity for prolonged follow-up and monitoring [12, 13]. Burn survivors with depressive symptoms face prolonged hospitalization, greater need for surgical and reconstructive interventions, increased medical care requirements, and elevated risk of suicidal ideation [14, 15]. Although the risk of depression rises following burn injuries, the specific factors contributing to its development remain unclear. Identifying these risk factors could enhance both the functional and psychological outcomes for burn survivors [16].

Numerous studies have examined risk factors for depression among burn survivors; however, prior reviews remain inconclusive due to the absence of quantitative synthesis [11, 17, 18]. Accordingly, this systematic review and meta-analysis aimed to evaluate the risk of depression among burn injury survivors, stratified by severity. Additionally, we assessed the potential risk factors contributing to post-burn depression. This information is essential for identifying patients at elevated risk and implementing timely preventive interventions to optimize outcomes.

## Materials and methods

We used The Preferred Reporting Items for Systematic Reviews and Meta-Analysis (PRISMA) guidelines [19] and the recommendations of the Cochrane collaboration [20] while conducting the present meta-analysis (Supplementary Table 1). We registered the protocol of the present review at the International Prospective Register of Systematic Reviews database (**CRD42023440045**).

We searched twelve databases from inception to 14 June 2023. This included PubMed, SIGLE, Google Scholar, Virtual Health Library (VHL), Web of Science (ISI), NYAM, Scopus, Clinical trials, EMBASE, Controlled Trials (mRCT), Cochrane Collaboration, and WHO International Clinical Trials Registry Platform (ICTRP). We performed an updated search of these databases on 29 September 2024. We used the following keywords customized for each database: ‘Depression’, ‘Depressed’, ‘Depressive’, ‘Mood Disorder’, ‘Psychology’, ‘Psychiatry’, ‘Burn’, ‘Burnt’, ‘Burned’. We performed further manual search, including cross-referencing, citation tracking, and reviewing the references of the included studies. This was carried out to include all additional relevant articles that were not identified through the searching process. The search strategy for each database is reported in Supplementary Tables 2 and Supplementary Table 3.

We included all clinical studies comparing the demographic, burn-related, or management-related factors between patients with depression and patients without depression among burn injury survivors. We excluded studies including patients with self-inflicted burn injuries, and patients with pre-burn depression. There was no restriction on the patient’s age, sex, race, type of burn injury, or place. We excluded non-comparative studies, guidelines, cadaveric studies, non-human studies, case reports, review articles, comments, letters, posters, editorials, and book chapters. We exported the relevant articles to the Excel sheet after removing the duplicates using EndNote X9 [21]. Two reviewers independently performed the title, abstract, and full-text screening process to determine the articles that were eligible for data extraction and analysis. We dissolve the contradictory findings by discussion.

We extracted the data in a well-formulated Excel sheet. We extracted the study characteristics, including the title of the included study, the second name of the first author, year of publication, study design, study period, and study region. We extracted the methodology-related data, including the eligibility criteria, timing of depression assessment, and depression criteria. We extracted the patients’ demographic characteristics, including the sample size, age, gender, weight, comorbidities, marital status, occupational status, and education level. We extracted the burn-related data, encompassing the type, degree, site, extent, cause, total body surface area, and time since injury. We retrieved the number of patients with depression and the degree of depression. We assessed the quality of the analyzed observational articles using the National Institute of Health (NIH) quality assessment tool [22].

We estimated the prevalence of depression among burn injury survivors by calculating the event rate and 95% confidence intervals (CIs) for each study. We then pooled the effect sizes across all studies to determine the overall proportion with 95% CI. We used the risk ratio (RR) with 95% CI to analyze dichotomous variables. We applied a fixed-effect model when assuming a fixed population effect size; otherwise, we used a random-effects model. We assessed statistical heterogeneity using the Higgins I² statistic (>50%) and the Cochrane Q (Chi² test, *p* < 0.10 [23]. We evaluated publication bias by examining asymmetry in the funnel plot and calculated it using Egger’s regression test (*P* < 0.10). We performed all analyses using RevMan 5.4 and Comprehensive Meta-Analysis v3 software. We executed data analysis using RevMan5.4 and Comprehensive Meta-Analysis v3 software [24, 25]. The significance was established at the value of (*P* < 0.05).

## Results

**Fig. 1:**
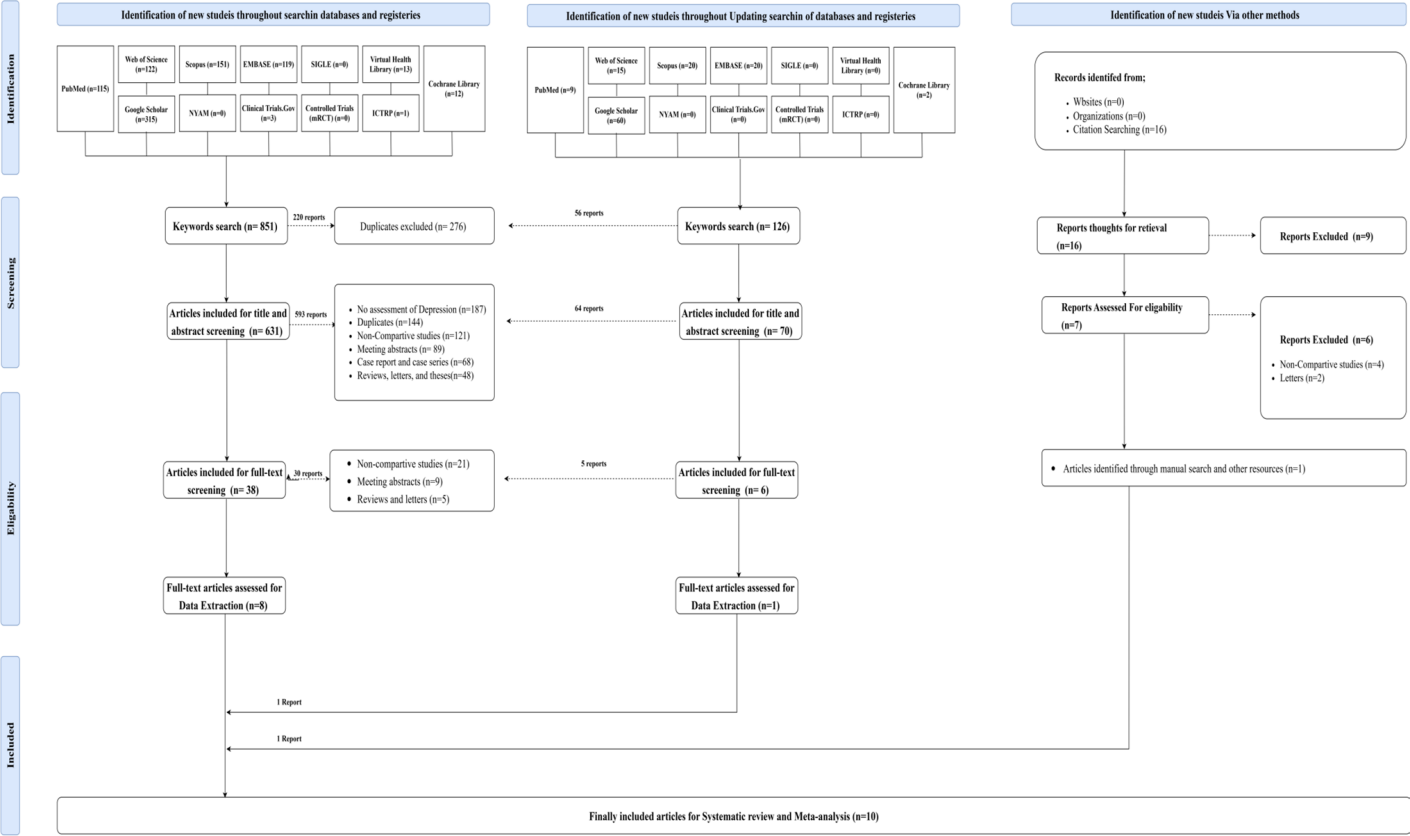
PRISMA 2020 flow diagram for the systematic review on the risk of depression among burn injury survivors, showing database, register, and other source searches, and the screening process

A systematic review identified 977 articles, of which 44 were eligible for full-text review. Following exclusion of 35 studies, nine articles were included for data extraction. One additional study was identified through manual searching, yielding a total of ten studies for systematic review and meta-analysis (Fig. 1).

The current meta-analysis included ten articles, encompassing 2957 patients with burn injuries [26–35]. Four studies used the Beck’s Depression Inventory scale for depression assessment among burn injury survivors. Of the 2,957 patients, 36.8% experienced depression, with 33.1% classified as mild, 34.0% as moderate, and 10.8% as severe. Age distribution included 15.2% under 20 years, 16.0% between 20 and 40 years, and 6.1% over 40 years. All studies were of high quality, with scores ranging from 80% to 90% (Table 1).

**Table 1:** Demographic characteristics and quality assessment of studies included in the systematic review on depression among burn injury survivors

Study ID		Study Region	Study Design	Study period	Assessment of depression	Sample Size		Degree of depression			Total Sample	Age (Years)	
						Depression	No-Depression	Mild	Moderate	Severe		<20	
												Depression	No-Depression
						Number	Number	Number	Number	Number	Number	Number	Number
1	Ahrari et al., 2013 [34]	Iran	Cross-sectional	April 2010 to May 2011	BDI II	184	116	58	52	74	300	13	17
2	Ali et al., 2019 [29]	Pakistan	Cross-sectional	September 2017 to February 2018	BDI II	30	64	15	8	7	94	0	0
3	Ammar et al., 2019[33]	Iraq	Case control	1 st of March 2017 to 1 st of September 2017	DSM-V	100	20	36	44	20	120	NR	NR
4	Asif et al., 2020[31]	Pakistan	Cross-sectional	17th August 2017 to 23rd August 2018	HADS	88	62	NR	NR	NR	150	0	0
5	Campbell et al., 1987[28]	USA	Cross-sectional	1977 to 1982	DSM-III	34	252	NR	NR	NR	286	34	252
6	Hamzawi et. al. 2018 [30]	Iraq	Case control	April 2nd 2014 to January 2nd 2015	ICD-10 and BDI II	40	60	12	24	4	100	20	25
7	Shahid et al., 2018[26]	Pakistan	Cross-sectional	March to August 2016	EQ-5D	163	78	NR	139	24	240	104	51
8	Rehan et al., 2024[32]	Pakistan	Prospective cohort	January to June 2022	HAM-D	200	4	99	68	38	204	NR	NR
9	Roh et al., 2012[36]	South Korea	Cross-sectional	NR	Korean CESD	56	57	NR	NR	NR	113	NR	NR
10	Thombs et al., 2007[35]	USA	Prospective cohort	December 1993 through May 1997	BDI II	193	69	140	35	10	262	NR	NR

Study ID		Age (Years)				Gender				Single Status		Quality assessment	
		20–40		>40		Males		Females		Depression	No-Depression		
		Depression	No-Depression	Depression	No-Depression	Depression	No-Depression	Depression	No-Depression				
		Number	Number	Number	Number	Number	Number	Number	Number	Number	Number	%	Decision
1	Ahrari et al., 2013 [34]	123	47	49	52	89	61	95	55	54	37	80%	Good
2	Ali et al., 2019 [29]	25	54	5	10	20	38	10	26	12	28	80%	Good
3	Ammar et al., 2019[33]	NR	NR	NR	NR	NR	NR	NR	NR	36	6	90%	Good
4	Asif et al., 2020[31]	60	57	28	5	55	36	33	26	NR	NR	80%	Good
5	Campbell et al., 1987[28]	0	0	0	0	NR	NR	NR	NR	NR	NR	80%	Good
6	Hamzawi et. al. 2018 [30]	20	28	0	7	15	25	25	35	NR	NR	90%	Good
7	Shahid et al., 2018[26]	38	20	21	7	68	37	95	41	NR	NR	80%	Good
8	Rehan et al., 2024[32]	NR	NR	NR	NR	107	3	89	1	NR	NR	90%	Good
9	Roh et al., 2012[36]	NR	NR	NR	NR	30	50	26	7	NR	NR	90%	Good
10	Thombs et al., 2007[35]	NR	NR	NR	NR	142	42	51	27	NR	NR	90%	Good

Abbreviations; *BDI* Beck's Depression Inventory, *DSM *Diagnostic and Statistical Manual of Mental Disorders, *HADS *Hospital Anxiety and Depression Scale, *ICD *International Classification of Disease, *E2Q *emotional quotient, *CESD *Center for Epidemiological Studies-Depression Scale, *NR *Non-reported

Regarding burn characteristics, 5.6% of patients sustained accidental burns. Second- and third-degree burns were observed in 10.7% and 4.1% of patients, respectively. Total body surface area < 30% was noted in 15.3% of patients with depression and 8.2% without. Burn types included thermal (23.6%), chemical (1.7%), and electrical (1.5%). Anatomically, burns affected the head and neck among 9.1% patients, and upper extremities among 10.2% patients (Table 2).

**Table 2:** Burn-related characteristics of patients in studies included in the systematic review on depression among burn injury survivors

Study ID		Causes of burn				Degree of burn				Total Body Surface area%		
		Combat related		Accidental		Second		Third		0–30		>30
		Depression	No-Depression	Depression	No-Depression	Depression	No-Depression	Depression	No-Depression	Depression	No-Depression	Depression
		Number	Number	Number	Number	Number	Number	Number	Number	Number	Number	Number
1	Ahrari et al., 2013 [34]	NR	NR	NR	NR	NR	NR	NR	NR	116	93	57
2	Ali et al., 2019 [29]	NR	NR	NR	NR	NR	NR	NR	NR	20	51	10
3	Ammar et al., 2019[33]	52	16	48	4	44	14	26	2	68	18	32
4	Asif et al., 2020[31]	NR	NR	NR	NR	NR	NR	NR	NR	NR	NR	NR
5	Campbell et al., 1987[28]	NR	NR	14	101	NR	NR	NR	NR	NR	NR	NR
6	Hamzawi et. al. 2018 [30]	NR	NR	NR	NR	NR	NR	NR	NR	NR	NR	NR
7	Shahid et al., 2018[26]	NR	NR	NR	NR	114	63	46	15	135	77	28
8	Rehan et al., 2024[32]	NR	NR	NR	NR	NR	NR	NR	NR	112	4	84
9	Roh et al., 2012[36]	NR	NR	NR	NR	40	41	16	16	NR	NR	NR
10	Thombs et al., 2007[35]	NR	NR	NR	NR	NR	NR	NR	NR	NR	NR	NR

Study ID		Total Body Surface area%	Type of Burn						Location of Burn			
		>30	Thermal		Chemical		Electrical		Head and Neck		Upper Extremity	
		No-Depression	Depression	No-Depression	Depression	No-Depression	Depression	No-Depression	Depression	No-Depression	Depression	No-Depression
		Number	Number	Number	Number	Number	Number	Number	Number	Number	Number	Number
1	Ahrari et al., 2013 [34]	15	NR	NR	NR	NR	NR	NR	NR	NR	NR	NR
2	Ali et al., 2019 [29]	13	19	39	7	9	4	16	5	5	6	18
3	Ammar et al., 2019[33]	2	88	20	6	0	6	0	NR	NR	NR	NR
4	Asif et al., 2020[31]	NR	88	62	NR	NR	NR	NR	NR	NR	NR	NR
5	Campbell et al., 1987[28]	NR	32	185	1	26	NR	NR	12	120	18	159
6	Hamzawi et. al. 2018 [30]	NR	NR	NR	NR	NR	NR	NR	NR	NR	NR	NR
7	Shahid et al., 2018[26]	1	NR	NR	NR	NR	NR	NR	NR	NR	NR	NR
8	Rehan et al., 2024[32]	0	NR	NR	NR	NR	NR	NR	NR	NR	NR	NR
9	Roh et al., 2012[36]	NR	NR	NR	NR	NR	NR	NR	32	27	NR	NR
10	Thombs et al., 2007[35]	NR	144	52	NR	NR	13	4	47	21	70	31

Abbreviations; NR=Non-reported

**Fig. 2:**
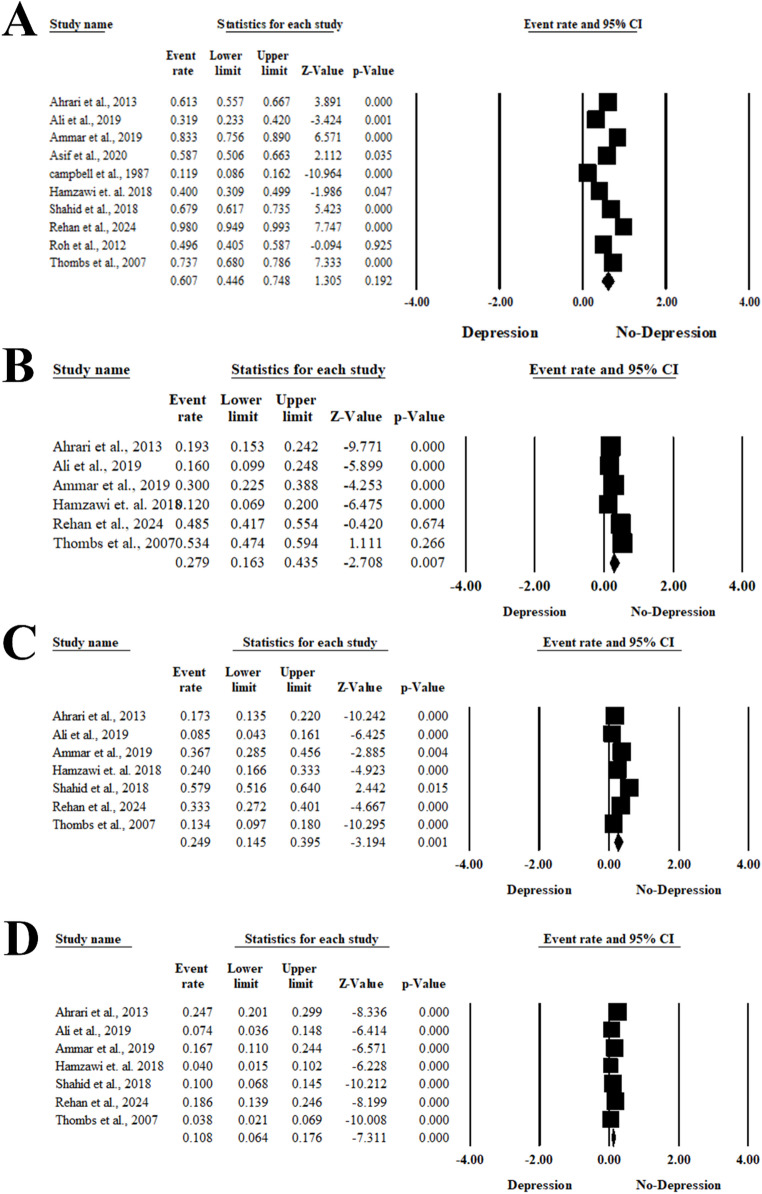
Forest plot of the overall prevalence of depression among burn injury survivors. (**A**) Event rate and 95% CI of overall depression. (**B**) Mild depression. (**C**) Moderate depression. (**D**) Severe depression. Black squares indicate the statistical weight of each study, and the grey diamond represents the pooled estimate. Positioning beyond the vertical line (unit value) indicates a significant outcome

Ten studies included 1869 patients evaluated the risk of depression among burn injury survivors [26–35]. Meta-analysis using a random-effects model (I² = 97.14%, *P* < 0.001) indicated a pooled depression prevalence of 60.7% (95% CI: 44.5–74.8%) among burn injury survivors. No evidence of publication bias was observed, as indicated by the symmetrical funnel plot and Egger’s regression test (Intercept = 1.296, *P* = 0.84). The risk of mild depression was reported within six articles, including 1080 patients [29, 30, 32–35]. Pooling data from six studies including 1,080 patients indicated a prevalence of mild depression of 27.9% (95% CI: 16.3–43.5%, *P* = 0.007). Seven studies comprising 1,320 patients evaluated moderate and severe depression among burn injury survivors [29, 30, 32–35]. The risk of moderate depression was 24.9% (95%CI; 14.5%- 39.5%, *P* = 0.001), and the risk of severe depression was 10.8% (95%CI; 6.4%−17.6%, *P* < 0.001) (Fig. 2A, B, C, and D).

## Factors Associated with Depression among Burn Injury Survivors

### Patient-related Factors

#### Age

**Fig. 3:**
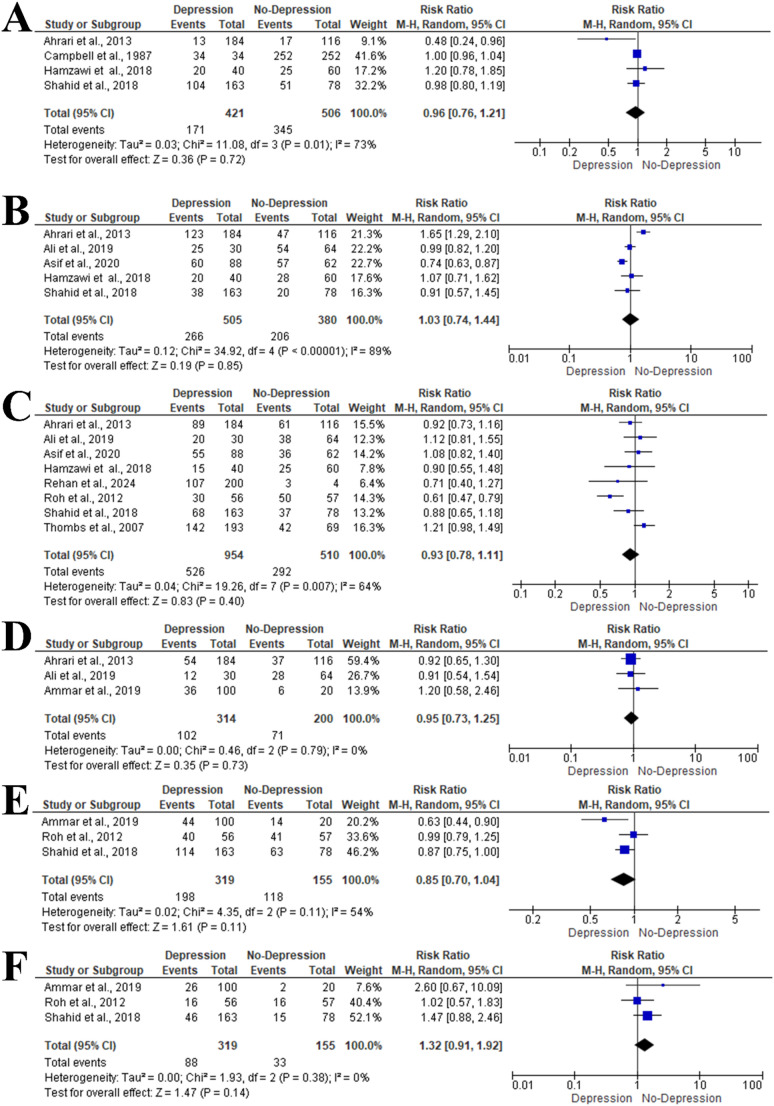
Forest plot of risk ratios (RR) and 95% CIs for factors associated with depression among burn injury survivors: (**A**) Age < 20 years, (**B**) Age 20–40 years, (**C**) Gender, (**D**) Marital status, (**E**) Second-degree burns, (**F**) Third-degree burns. Blue squares indicate study weight; grey diamonds show pooled estimates; positioning beyond the vertical line indicates significance (IV = inverse variance)

Four articles included 927 patients with burn injuries evaluated the influence of age (< 20 years) on the risk of depression [26, 28, 30, 34]. Meta-analysis using a random-effects model (I² = 73%, *P* = 0.01) showed no significant difference between depression and non-depression groups (RR = 0.96, 95% CI: 0.76–1.21, *P* = 0.72). Similarly, age 20–40 years had no significant impact on depression risk (RR = 1.03, 95% CI: 0.74–1.44, *P* = 0.85; I² = 89%, *P* < 0.001) (Fig. 3A and B).

## Gender

The impact of male gender on the risk of depression was reported in eight articles including 1464 patients [26, 27, 29–32, 34, 35]. Meta-analysis using a random-effects model (I² = 64%, *P* = 0.007) showed no significant association (RR = 0.93, 95% CI: 0.78–1.11, *P* = 0.40) (Fig. 3C).

### Marital Status

Three studies included 514 patients assessing the influence of single status on the risk of depression among burn injury survivors [29, 33, 34]. Meta-analysis using a random-effects model (I² = 0%, *P* = 0.79) showed no significant difference between depression and non-depression groups (RR = 0.95, 95% CI: 0.73–1.25, *P* = 0.73) (Fig. 3D).

## Burn-related Factors

### Degree of Burn

Three studies included 474 patients evaluated the risk of depression among patients with second and third-degree burns [26, 27, 33]. Meta-analysis showed no significant impact of second-degree burns (RR = 0.85, 95% CI: 0.70–1.04, *P* = 0.11) or third-degree burns (RR = 1.32, 95% CI: 0.91–1.92, *P* = 0.14) on depression risk among burn injury survivors (Fig. 3E and F).

### Total Body Surface Area (%)

**Fig. 4:**
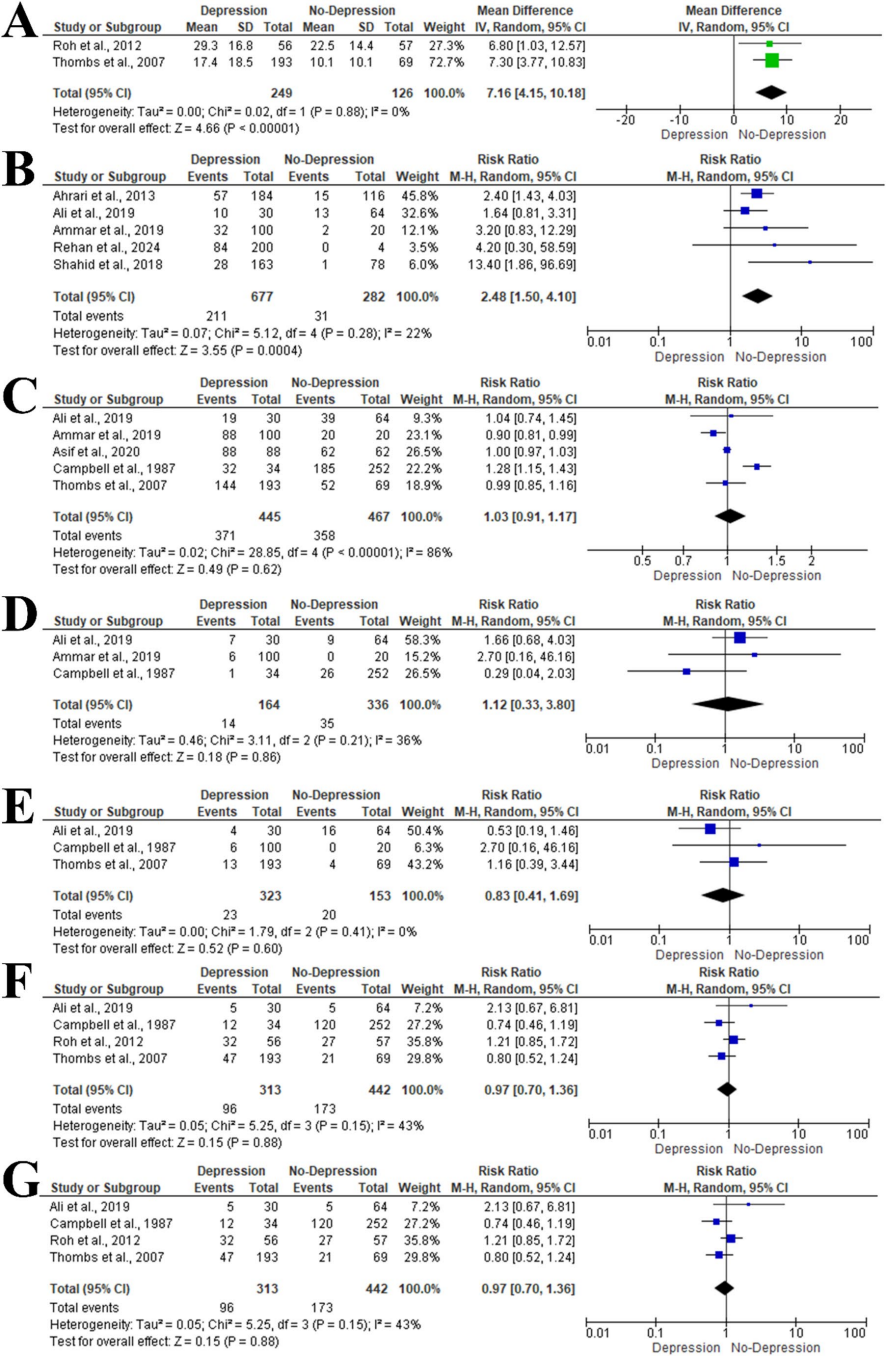
Forest plot of additional risk factors for depression among burn injury survivors: (**A**) Mean difference in total body surface area (TBSA) % between patients with and without depression, (**B**) TBSA > 30%, (**C**) Thermal burn injury, (**D**) Gender, (**E**) Chemical burn injury, (**F**) Electrical burn injury, (**G**) Facial burn injury, (**H**) Upper limb burn injury. Green/blue squares indicate study weight; grey diamonds represent pooled estimates; positioning beyond the vertical line indicates significance (IV = inverse variance)

Two studies included 375 patients evaluated the difference between depression and non-depression groups regarding the mean total body surface area % [27, 35]. Meta-analysis using a random-effects model (I² = 0%, *P* = 0.88) showed a significantly higher mean total body surface area % in patients with depression (mean difference = 7.16, 95% CI: 4.15–10.18, *P* < 0.001). Five studies including 959 patients assessed the impact of total body surface area >30% on depression risk, revealing a 2.48-fold increased risk (RR = 2.48, 95% CI: 1.50–4.10; I² = 22%, *P* = 0.28) (Fig. 4A and B).

### Type of Burn

Five reports included 912 patients evaluated the impact of thermal burn injury on the risk of depression [28, 29, 31, 33, 35]. Meta-analysis showed no significant association (RR = 1.03, 95% CI: 0.91–1.17; I² = 86%, *P* < 0.001). Similarly, chemical burns (RR = 1.12, 95% CI: 0.33–3.80, *P* = 0.86) and electrical burns (RR = 0.83, 95% CI: 0.41–1.69, *P* = 0.60) were not significantly associated with depression (Fig. 4C and D, and E).

### Location of Burn

Four studies including 755 patients evaluated depression risk in individuals with facial burns [27–29, 35]. Meta-analysis using a random-effects model (I² = 43%, *P* = 0.15) showed no significant association (RR = 0.97, 95% CI: 0.70–1.36, *P* = 0.88) (Fig. 4F). The effect of upper limb burns was assessed in three studies including 642 patients [28, 29, 35], with no significant impact on depression risk (RR = 0.81, 95% CI: 0.65–1.02, *P* = 0.07; I² = 0%, *P* = 0.93) (Fig. 4G).

## Discussion

This meta-analysis demonstrated that approximately 60.7% of burn injury survivors experience depression, with higher rates observed among those with major burns. Patients with total body surface area >30% were approximately 2.5 times more likely to develop depression, whereas age, gender, marital status, burn type, burn degree, and burn site had no significant effect. These findings emphasize that depression should be anticipated in burn survivors and underscore the need for standardized psychological care. More than half of the patients experienced depressive symptoms, predominantly of mild to moderate severity, while approximately 10% suffered severe depression. These results align with previous findings by Thombs et al. 2006, who reported a 4–10% prevalence of major depression among burn patients [18]. Woolard et al., 2021 reported an increase in anxiety and psychological insults following burn injury, particularly among children and adolescents [37]. A significant association was observed between burn extent and depression risk. Similarly, Mehrabi et al. 2022 reported that burn severity, including percentage, depth, and facial involvement was negatively associated with self-esteem and positively correlated with major depression among burn survivors [36]. Major burns cause extensive damage to the skin and underlying tissues, often resulting in substantial cosmetic and functional impairment. The associated scarring and altered appearance can significantly affect self-esteem and body image. Moreover, recovery typically involves prolonged and demanding physical and psychological rehabilitation, which is frequently stressful and painful. Burn survivors are also at increased risk of PTSD and social anxiety, both of which are important contributors to depression [38–40]. Depression may intensify the disability and the burden of burn injury, complicate the recovery, and deteriorate the long-term functional outcomes. Spronk et al., 2018 reported the negative impact of burn severity and postburn depression on quality of life among burn injury survivors [41].

Depression among burn survivors underscores the need for timely screening and intervention. Burn management should support both psychological and physical recovery, minimize functional impairment, facilitate social reintegration, and enhance overall quality of life. Psychological support should be integrated into burn care programs as a standard component to address behavioral and emotional barriers and optimize psychosocial rehabilitation [42]. Developing psychological interventions tailored to each patient’s needs and unique risks is paramount. Such interventions could ultimately improve the well-being and prognosis of burn injury survivors [17]. Wang et al., 2024 emphasized the complexity of addressing the psychological insults among burn injury survivors and highlighted the need to develop more standardized care for such a population [43].

This systematic review confirmed a substantial risk of depression among burn survivors. However, several limitations should be considered. Most included studies were cross-sectional, which may introduce selection and information bias. Significant heterogeneity was observed, likely due to differences in inclusion criteria, patient demographics, depression assessment methods, burn types, and geographic settings. A random-effects model was applied to account for this heterogeneity.

Depression affects approximately two-fifths of burn survivors, with one-quarter experiencing moderate depression and one-tenth severe depression, especially in major burns. Age, gender, marital status, burn type, severity, and site had minimal impact. These findings highlight the need for systematic psychological assessment and standardized interventions to optimize recovery and quality of life in this population.

## Supplementary Information

Below is the link to the electronic supplementary material.

Supplementary Material 1

## Data Availability

Yes. The datasets used in the present study are available from the first author and corresponding authors on reasonable request.

## Declarations

### Level of Evidence

Level III.

## Abbreviations

PTSD: post-traumatic stress disorder
PRISMA: The Preferred Reporting Items for Systematic Reviews and Meta-Analysis
NIH: National Institute of Health
CI: confidence intervals
RR: risk ratio
